# Relearning functional and symmetric walking after stroke using a wearable device: a feasibility study

**DOI:** 10.1186/s12984-019-0569-x

**Published:** 2019-08-28

**Authors:** Seok Hun Kim, David E. Huizenga, Ismet Handzic, Rebecca Edgeworth Ditwiler, Matthew Lazinski, Tyagi Ramakrishnan, Andrea Bozeman, David Z. Rose, Kyle B. Reed

**Affiliations:** 1 University of South Florida, School of Physical Therapy and Rehabilitation Sciences, Tampa, FL USA; 2 Moterum Technologies Inc., Greenville, SC USA; 3 Northern New Mexico College, Espanola, NM USA; 4 University of South Florida Department of Neurology, Tampa, FL USA; 5 University of South Florida, Department of Mechanical Engineering, Tampa, FL USA

**Keywords:** Stroke, Rehabilitation, Asymmetry, Hemiparetic gait

## Abstract

**Background:**

Gait impairment is a common consequence of stroke and typically involves a hemiparetic or asymmetric walking pattern. Asymmetric gait patterns are correlated with decreased gait velocity and efficiency as well as increased susceptibility to serious falls and injuries.

**Research Question:**

This paper presents an innovative device worn on a foot for gait rehabilitation post stroke. The device generates a backward motion to the foot, which is designed to exaggerate the existing step length asymmetry while walking over ground. We hypothesize this motion will decrease gait asymmetry and improve functional walking in individuals with chronic stroke.

**Methods:**

Six participants with chronic stroke, more than one year post stroke, received four weeks of gait training with three sessions per week. Each session included 30 min of walking over ground using the wearable device. Gait symmetry and functional walking were assessed before and after training.

**Results:**

All participants improved step length symmetry, and four participants improved double limb support symmetry. All participants improved on all three functional outcomes (gait velocity, Timed Up and Go Test, and 6-Minute Walk Test), and five participants improved beyond the minimal detectable change or meaningful change in at least one functional outcome.

**Conclusion:**

The results indicate that the presented device may help improve stroke patients’ walking ability and warrant further study. A gait training approach using this new device may enable and expand long-term continuous gait rehabilitation outside the clinic following stroke.

**Trial registration:**

NCT02185404. Registered July 9, 2014, https://clinicaltrials.gov/ct2/show/NCT02185404

## Introduction

Each year approximately 800,000 Americans experience a new or recurrent stroke, and an estimated six million are living with gait impairments from a stroke [1]. One such disability is a ‘hemiparetic’ gait [2], which can be characterized by asymmetries in gait measures such as step length and support times [3, 4]. Hemiparetic gait is correlated with decreased gait velocity [5, 6], reduced walking efficiency [7], increased joint and bodily degradation [8], and increased susceptibility to injuries and falls [9, 10].

While patients and health providers desire effective gait therapy, few effective long-term remedies have been identified. Treatments of gait commonly rely on traditional rehabilitation approaches, such as the Bobath method [11, 12] and lower limb strength training [13, 14], to re-train walking patterns. Unfortunately, results are inconsistent across patient populations with these treatment options, and there are not set devices facilitating these treatments. Some other gait correction methods currently being studied include Constraint Induced Movement Therapy [15, 16], body-weight support [17], robotic [18], functional electrical stimulation [19], transcranial magnetic stimulation [20], and full-body gait exoskeletons [21].

**Fig. 1:**
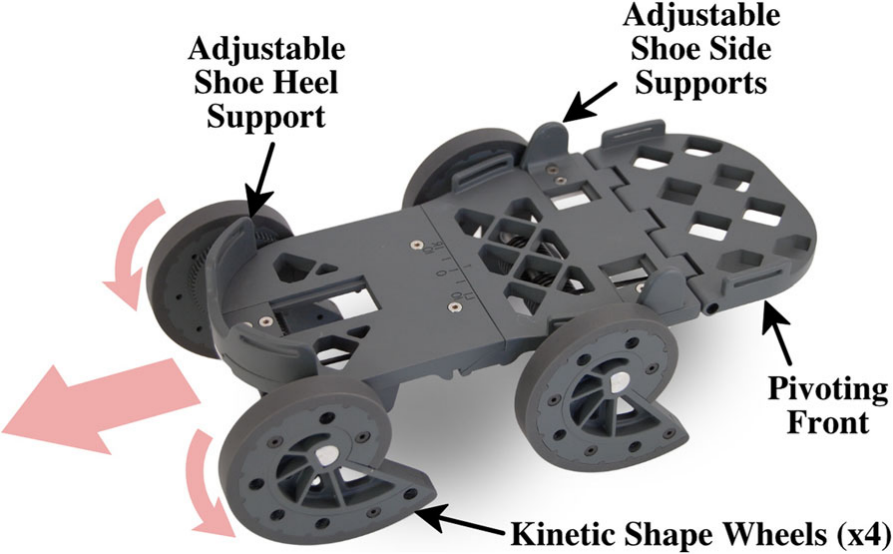
Photo of the rehabilitative shoe that is worn on the nonparetic foot

**Fig. 2:**
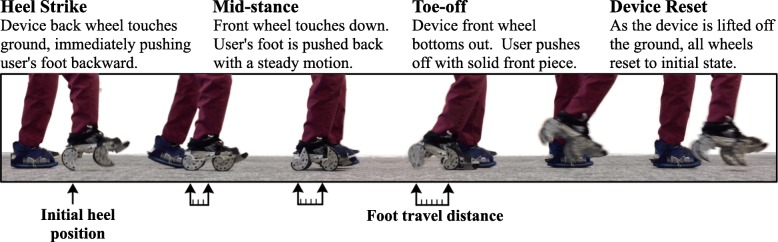
As the wearer takes a step, the device pushes the nonparetic foot backward during stance. This exaggeration of the step length asymmetry is hypothesized to result in a more symmetric gait pattern once the shoe is removed. In addition, the shoe works to strengthen the paretic leg by slightly destabilizing the nonparetic leg, which encourages the wearer to use their paretic leg more. A flexible height and weight matched platform worn on the paretic foot equalizes the added height and weight of the device

In this paper, we present a novel device (shown in Fig. 1) designed to help individuals post stroke re-learn how to walk with little to no therapeutic infrastructure needed. Unlike many of the existing gait rehabilitation devices, this device is passive, portable, wearable, and does not require any external energy. It functions by moving the nonparetic foot backward while the individual walks over ground [22]. The backward motion of the shoe is generated passively by redirecting the wearer’s downward force during stance phase [23]. Since the motion is generated by the wearer’s force, the person is in control, which allows easier adaptation to the motion, but this also causes the speed to vary slightly from person to person. The generated motion is demonstrated in Fig. 2. A height and weight matched shoe is attached to the paretic foot, but does not generate any motion.

We hypothesize that this shoe helps with gait relearning in several ways. First, the backward motion exaggerates step length asymmetry such that some of the resulting spatiotemporal aspects of gait will be more symmetric once the shoe is taken off. This can be thought of as a version of error augmentation [18, 24] where the asymmetric step length is exaggerated. Secondly, the motion of the shoe encourages the use of the paretic side by making it harder to walk on the nonparetic side. Thirdly, it is untethered and portable, so it enables rehabilitation in a variety of locations. Making rehabilitation available in more locations should improve the context-dependent learning [25] so patients are relearning gait in the same places that they will generally be walking. This context should also help generalize the knowledge to real-world scenarios instead of just the laboratory setting. Fourthly, the device could enable patients to work on their rehabilitation with greater frequency and for a longer duration than currently available methods. Consistent, regular rehabilitation sessions have been shown to lead to enhanced rehabilitation effects [26]. Furthermore, neural repatterning is known to improve with just one session per sleep cycle through motor memory consolidation [27], and thus the increased ability to use the device daily should have beneficial learning effects compared to less frequent visits to a clinic. This feasibility study is conducted in the clinic to focus on the first two points. A future study to be done in participants’ own homes will evaluate the third and fourth points.

The shoe design has been presented previously [28, 29] along with evaluations on unimpaired participants [30], and a model of passive dynamics of walking was used to compare the shoe motions to a split-belt treadmill and normal walking [22]. This paper presents a feasibility study showing how the gait of six individuals with chronic stroke changed when using the device for 12 sessions over four weeks.

## Methods

### Participants

Details about the six participants recruited into the study are shown in Table 1. All participants signed a consent form that was approved by the Western Institutional Review Board prior to the study. Consent took place at the University of South Florida. Recruitment of participants occurred from 2015–2016. All experiments were completed by 2017.

**Table 1:** Characteristics of study participants

Participant	Age (years)	Gender	Height (cm)	Weight (kg)	Years Post Stroke	Type of Stroke	Paretic Limb
1	60	Male	185.4	111.1	4.5	Hemorrhagic	Left
2	74	Female	147.3	39.5	1.2	Ischemic	Left
3	63	Male	175.3	113.4	6.4	Hemorrhagic	Left
4	57	Female	159.0	70.8	5.2	Ischemic	Left
5	67	Male	180.3	78.9	10.8	Hemorrhagic	Right
6	73	Male	161.5	65.0	12.5	Ischemic	Right

Inclusion criteria included: (1) age 21–80, (2) one or more cerebral strokes, (3) a stroke at least six months prior to enrollment, (4) gait asymmetry greater than 2.5% step length or stance phase based on the pre-test, but able to walk either with or without a cane for at least five minutes, (5) no evidence of uncompensated hemianopsia, tested by using Pedretti’s method [31], (6) no evidence of severe (less than 16 in the Folstein Mini-Mental Status Exam) cognitive impairment [32] or language dysfunction that would interfere with understanding the instructions, and (7) no evidence of neglect, tested by being asked to copy a simple drawing of a house [33]. Exclusion criteria included: (1) orthopedic or pain conditions, (2) uncontrolled seizures, (3) metal implants (e.g., stents, clips, pacemaker), (4) pregnancy, (5) any condition that makes balance unstable, and (6) uncorrected visual impairments.

### Experimental setup

The study consisted of one baseline gait evaluation (pre-test) followed by four weeks of training on the rehabilitative shoe for three sessions per week. A post training gait evaluation (post-test) was conducted three to five days after the last training session. All trainings and tests were performed at the Tampa campus of the University of South Florida.

### Training sessions

Each of the 12 training sessions consisted of six bouts of walking while wearing the rehabilitative shoe for five minutes with approximately two minute breaks in between bouts. The device was attached to the participant’s nonparetic foot. A height and weight matched shoe was attached to the paretic foot. Each shoe was secured to the foot with Velcro straps. Figure 2 shows an example of a participant walking during the training session. A licensed physical therapist trained for walking assistance walked next to each participant and loosely held a gaitbelt that the participant was wearing during all training bouts to ensure participant safety. Vital signs (i.e., heart rate, blood pressure, respiratory rate, and oxygen saturation) were measured before the first training bout and after each bout of walking on the device to ensure participant safety and monitor their response. The modified Borg Rating of Perceived Exertion Scale [34] was also used as a self-reported subjective measure of physical exertion.

### Gait evaluations and data analysis

We evaluated three spatiotemporal gait measures and three functional measures of gait before and after training. An 8 meter ProtoKinetics Zeno Walkway System (ProtoKinetics LLC, Havertown, PA) measured the gait patterns during each evaluation. The participant was instructed to walk over the walkway five times at his/her comfortable gait velocity. The three functional measures included three repetitions of the Timed Up and Go (TUG) test, one 6-Minute Walk Test (6MWT), and gait velocity was measured during the five repetitions on the ProtoKinetics Zeno Walkway System. Note that participant 5 did not complete the TUG or 6MWT during the followup visit.

The percentage of asymmetry for the three gait measures (i.e., step length, stance phase, and double limb support) are calculated by:

1 $$ \text{\% of Asymmetry} \,=\, \frac{abs(\text{M\(_{paretic}\)} - \text{M\(_{nonparetic}\)})}{\,\frac{1}{2}*(\text{M\(_{paretic}\)}+\text{M\(_{nonparetic}\)})\,}*100\%   $$

where M is the measure, and a value of 0 indicates symmetry. Step length is calculated as the anterior-posterior distance between the heel centers of two successive feet specified based by which leg is leading. Stance phase is calculated as the percent of the gait cycle (i.e., between two successive heel strikes of the same foot) between heel strike and toe off of the same foot. Double limb support (DLS) phase is calculated as the percent of the gait cycle that both feet are touching the ground specified by which leg is leading. The asymmetry measures were determined during the five repetitions on the ProtoKinetics Zeno Walkway System. Descriptive analyses were used to identify the effects of gait training with the device on gait symmetry and functional outcomes.

## Results

The individual gait asymmetry measures are shown in Table 2. All six participants improved step length symmetry. Four participants improved DLS symmetry. Four participants improved stance phase symmetry although the amount of change was relatively small.

**Table 2:** Changes in gait asymmetry measures after training

Participant	Step Length Asymmetry (%)		Double Limb Support Asymmetry (%)		Stance Phase Asymmetry (%)	
	pre		pre		pre	
	post	change	post	change	post	change
1	19.5		29.4		14.5	
	13.8	-5.7	11.7	-17.7	14.9	0.4
2	7.0		39.1		16.2	
	4.5	-2.5	31.4	-7.7	13.4	-2.8
3	261.5		106.9		12.0	
	242.2	-19.3	108.7	1.8	9.1	-2.9
4	42.4		21.4		30.2	
	35.3	-7.1	19.6	-1.8	33.1	2.9
5	19.0		26.9		29.7	
	9.8	-9.2	18.7	-8.3	28.6	-1.1
6	5.4		9.4		3.0	
	3.8	-1.6	19.5	10.1	2.3	-0.7
**Averages**	**59.1**		**38.9**		**17.6**	
	**51.6**	**-7.5**	**34.9**	**-4.0**	**16.9**	**-0.7**

The individual functional measures are shown in Table 3. All participants improved on all three functional outcomes (gait velocity, TUG, and 6MWT). Four participants demonstrated larger than a small meaningful change in gait velocity (≥ 6 cm/s) [35]. Two of the participants’ improvements were larger than the minimal detectable change (MDC) in TUG (≤− 3.5 sec) [36]. Altogether, five of the participants improved more than at least one of these MDC or small meaningful change limits.

**Table 3:** Changes in functional measures after training

Participant	Gait Velocity (cm/s)		TUG (sec)		6MWT (m)	
	Pre		Pre		Pre	
	post	Change	post	Change	post	Change
1	41.4		27.4		144.0	
	45.9	4.5	26.5	-0.8	145.5	1.5
2	47.0		21.3		150.2	
	61.1	14.1	20.4	-1.0	182.6	32.4
3	9.0		105.4		33.6	
	12.8	3.8	90.7	-14.8	42.4	8.8
4	35.9		24.1		149.4	
	43.9	8.0	19.6	-4.6	161.1	11.7
5	38.6		28.6		137.9	
	46.0	7.4	N/A	N/A	N/A	N/A
6	113.5		11.7		404.2	
	145.1	31.6	10.5	-1.1	430.5	26.3
**Averages**	**47.5**		**38.0**		**176.3**	
	**59.1**	**11.6**	**33.5**	**-4.5**	**192.4**	**16.1**

## Discussion

### Asymmetry measures

Walking on the rehabilitative shoe may benefit gait symmetry. All participants improved step length symmetry after training, and the average change in step length symmetry found in our study is similar to that shown in a study focused on gait symmetry during split-belt treadmill (SBT) training [37]. Four participants improved DLS symmetry; the two that did not improve were the severely impaired participant (initial gait velocity of 9.0 cm/s) and the highly functional participant (initial gait velocity of 113.5 cm/s). Although these two did not respond with a DLS asymmetry change, participant 3 improved step length asymmetry and had a substantial decrease on the TUG, and participant 6 improved on the 6MWT and had a substantial increase in gait velocity. For comparison, related studies show no change in DLS symmetry following SBT training [37, 38]. Our results suggest that over-ground gait training using the rehabilitative shoe could provide an additional benefit to the recovery of DLS symmetry for some individuals after stroke.

The literature does not provide estimates of the clinical relevance of gait asymmetry measures while walking over ground, but does provide some spatiotemporal measures for treadmill walking [39]. However, gait asymmetry has been associated with balance [40, 41] and is considered a major cause of future degenerative issues with hips, knees, and backs for stroke survivors with gait hemiparesis [42, 43].

### Functional measures

Walking on the rehabilitative shoe may help individuals with hemiparetic stroke improve their functional walking. Two of the participants’ gait velocity increased beyond a substantial meaningful change (≥ 14 cm/s), two other participants’ gait velocity increased beyond a small meaningful change (≥ 6 cm/s), and the remaining two improved less than these ranges. These ranges are based on people 30 to 150 days post stroke [35, 44]. One participant improved gait velocity beyond the clinically meaningful change of ≥ 16 cm/s that another study reported for people less than 60 days post stroke [45]. All the participants in our study were more than one year post stroke, which is much greater than the groups reported in these studies. Another important measure is that two out of three participants who were initially categorized as household ambulators (i.e., gait velocity of < 40 cm/s) became limited community ambulators (i.e., gait velocity of 40–80 cm/s) after training [5]; these two participants were 5 and 10 years post stroke

All participants who were assessed improved on the TUG, and two of them improved beyond the MDC of − 3.5 sec [36]. Although all five participants assessed improved the distance walked in the 6MWT following training, none of them surpassed the smallest minimal clinically important difference (MCID) of 34 m reported by Fulk and He [46]. The four-week training with only 6 hours total walk time may not be long enough for each participant to show a meaningful change in aerobic capacity. Continued daily use of the device for a longer time coupled with concomitant exercise may help them further increase aerobic capacity over time.

### Subjective evaluations

At the conclusion of the training and post-testing, we discussed the device and therapy with each participant and family member (if present). All the participants were generally positive about it. One participant was very encouraged by the amount of improvement she had following the training. Her thoughts are summarized by the two following statements: “I walked into church last week without a cane for the first time [since my stroke].” Her husband followed up by stating: “Her confidence walking around the house has increased dramatically since she started walking on the shoe.” Another participant stated: “I am able to walk faster and my knee moves and my toes have started to move. And those are a couple of things that didn’t happen before.” A video interview with one of the participants is available [47].

### Therapeutic mechanisms

The device presented is unlike any known existing rehabilitation therapies and is thought to function through a combination of mechanisms. These mechanisms likely benefit each individual uniquely since stroke presents in different ways. For example, all the participants showed a shorter stance phase with the paretic side compared to the nonparetic side, but three of the participants (1, 2, & 6) had a shorter step length with the paretic side. Although these three participants showed a smaller improvement in step length asymmetry, participant 1 showed the largest double limb support asymmetry improvement and participants 2 and 6 showed the largest gait velocity improvements. Encouraging more use of the paretic foot likely had a larger benefit to these participants. These unique benefits suggest that our device may have a heterogenous set of mechanisms that can benefit a wide set of stroke patients’ specific gait impairments. Further, all of our participants benefitted from this treatment, which is different than some of the SBT studies that show no gait symmetry improvements, especially step length symmetry, in approximately 40% of participants [37, 38]. Below are details on some the mechanisms we believe cause our device to help correct gait.

**Asymmetric Motion:** Both the presented device and the SBT cause one foot to move backward faster than the other. In SBT training, the gait asymmetry of the patient is increased by having two treads move at different speeds so that the patient must compensate to stay moving on the treadmill. When the belts are returned to the same speed, the patient will retain the “adjusted”, now more symmetric, gait on the treadmill [37, 48]. Our presented device moves the foot backward relative to the paretic foot, much like the motion of the fast tread of a SBT. Both the SBT and our device beneficially change step length symmetry, but only our device shows improvements in double limb support symmetry. This additional gait benefit is likely due the device attaching to the foot, which allows training in an over-ground context.

**Context Awareness:** The corrected walking patterns from existing therapeutic methods, such as treadmills, do not completely transfer to over-ground walking because the dynamic and sensorimotor aspects of walking over ground are distinctly different than walking on a treadmill [22, 28, 49, 50]. Research has indicated that only about 60% of the gait correction from walking on a split-belt treadmill transfers to walking over ground in individuals with stroke [51].

When walking over ground, an individual has complete control over velocity, whereas the treadmill speed limits one’s ability to change velocity. Another important difference is the amount of visual flow: on a treadmill, the scene is not moving, so there are no visual cues reinforcing the forward motion that would be present when walking over ground. Since walking is highly context dependent [25, 51–54], these visual cues indicating a different context may prevent the learned patterns on the treadmill from being expressed during over-ground walking. Our device allows over-ground walking in the environment of daily activities. A user of our device experiences a congruent dynamic optical/visual flow as opposed to an individual on a SBT, who typically views a static scene that is incongruent to training movements.

**Cueing:** The benefits of this device may also arise from the multiple cues produced by the device that guide the user through their gait. The first cue is that the nonparetic foot height is decreasing after first contact in stance; a second cue is that the nonparetic foot begins moving backward during the transition to stance. These cues start before the paretic leg transitions from stance, which provides a set of cues that possibly indicate the type of step to take with the paretic leg. For example, the first cue may induce more weight bearing on the paretic leg at mid-stance, while the second cue may foster earlier toe off of the paretic leg at terminal stance.

**Encouraging Paretic Leg:** The device can also increase the relearning of the paretic leg by reducing the effective output of the nonparetic leg by generating a backward motion. The motion induced by the device encourages the wearer to increase the use of their paretic leg. This effect is similar to the idea of Constraint Induced Movement Therapy [15, 16]. By slightly destabilizing their nonparetic leg, the user will naturally start to spend more time on their paretic side, which may help to foster those abilities and confidence in using that side of their body.

**Home Rehabilitation:** The literature has continued to show that patients are dissatisfied with their options for training after they are discharged from the rehabilitation hospital/clinic [55–59]. Moreover, most individuals with stroke prefer a home-based approach for their initial rehabilitation [60]. The ability to train at home enables individuals to more frequently rehabilitate themselves, which leads to better results in motor relearning [61] and can maintain individuals’ ability to perform activities of daily living [62, 63]. Our device has the potential to be used in the home setting, which could reduce the costs and increase the access as well as the amount of rehabilitation.

There are open questions related to the frequency of training, the length of each session, and how many weeks the training should continue. The intensity of the training during each session can also be customized by adjusting the spiral wheel to make the generated backward motion longer and/or faster. This customization could also be adjusted at regular intervals to keep a constant intensity level. Future studies will evaluate how to optimize the therapy further.

Safety is vital, particularly during home care. Using the device independently in a safe way is being evaluated in a separate home-based trial. In the study presented here, we found that participants became comfortable with the device within the first three sessions and needed little or no assistance after that. Out of the over 400 bouts of walking in our study, the attending PT only provided physical assistance twice due a perceived need for patient support. As such, we expect that home-based therapy could be provided for many patients after they complete a few sessions in the clinic and become qualified for home-use. The specific requirements of being eligible for home-use are being evaluated and will be discussed further once the larger home-based study is complete.

### Limitations

A limitation to this study is that only six participants were evaluated. A study with a larger sample size will provide more details about how these effects generalize across different stroke gait patterns. Another limitation is that this study did not have a matched control group to compare to standard physical therapy or simply walking for a similar amount of time [64]. Despite these limitations, the initial results are promising and suggest that further study is warranted.

## Conclusions

The presented gait training device was tested on six individuals with chronic stroke for twelve sessions over four weeks. During this time, all participants improved on step length symmetry, four improved on double limb support symmetry, and all improved on all three functional outcome measures. Five of the six participants improved beyond the MDC or meaningful change in at least one functional outcome. These results demonstrate the feasibility of this device to improve a chronic, hemiparetic stroke survivor’s gait symmetry and walking function. An additional study of this device will further understand the impact on the post stroke survivor’s quality of life, health range, future joint and musculature degeneration, and morale.

## Data Availability

Please contact author for data requests.
